# 100+ years of phase variation: the premier bacterial bet-hedging phenomenon

**DOI:** 10.1099/mic.0.001537

**Published:** 2025-02-27

**Authors:** Christopher D. Bayliss, Jack L. Clark, Marjan W. van der Woude

**Affiliations:** 1 Department of Genetics, Genomics and Cancer Sciences, University of Leicester, Leicester, UK; 2 Hull York Medical School and the York Biomedical Research Institute, University of York, York, UK

**Keywords:** bistability, contingency loci, instability, localised hypermutation, phase variation, phasevarion, phasome

## Abstract

Stochastic, reversible switches in the expression of *Salmonella* flagella variants were first described by Andrewes in 1922. Termed phase variation (PV), subsequent research found that this phenomenon was widespread among bacterial species and controlled expression of major determinants of bacterial–host interactions. Underlying mechanisms were not discovered until the 1970s/1980s but were found to encompass intrinsic aspects of DNA processes (i.e. DNA slippage and recombination) and DNA modifications (i.e. DNA methylation). Despite this long history, discoveries are ongoing with expansions of the phase-variable repertoire into new organisms and novel insights into the functions of known loci and switching mechanisms. Some of these discoveries are somewhat controversial as the term ‘PV’ is being applied without addressing key aspects of the phenomenon such as whether mutations or epigenetic changes are reversible and generated prior to selection. Another ‘missing’ aspect of PV research is the impact of these adaptive switches in real-world situations. This review provides a perspective on the historical timeline of the discovery of PV, the current state-of-the-art, controversial aspects of classifying phase-variable loci and possible ‘missing’ real-world effects of this phenomenon.

## Data availability

All data are either included within the manuscript or are openly available at the University of Leicester Research Repository (https://doi.org/10.25392/leicester.data.28437002.v1).

## Population heterogeneity and definition of the phase variation phenomenon

**Fig. 1.**
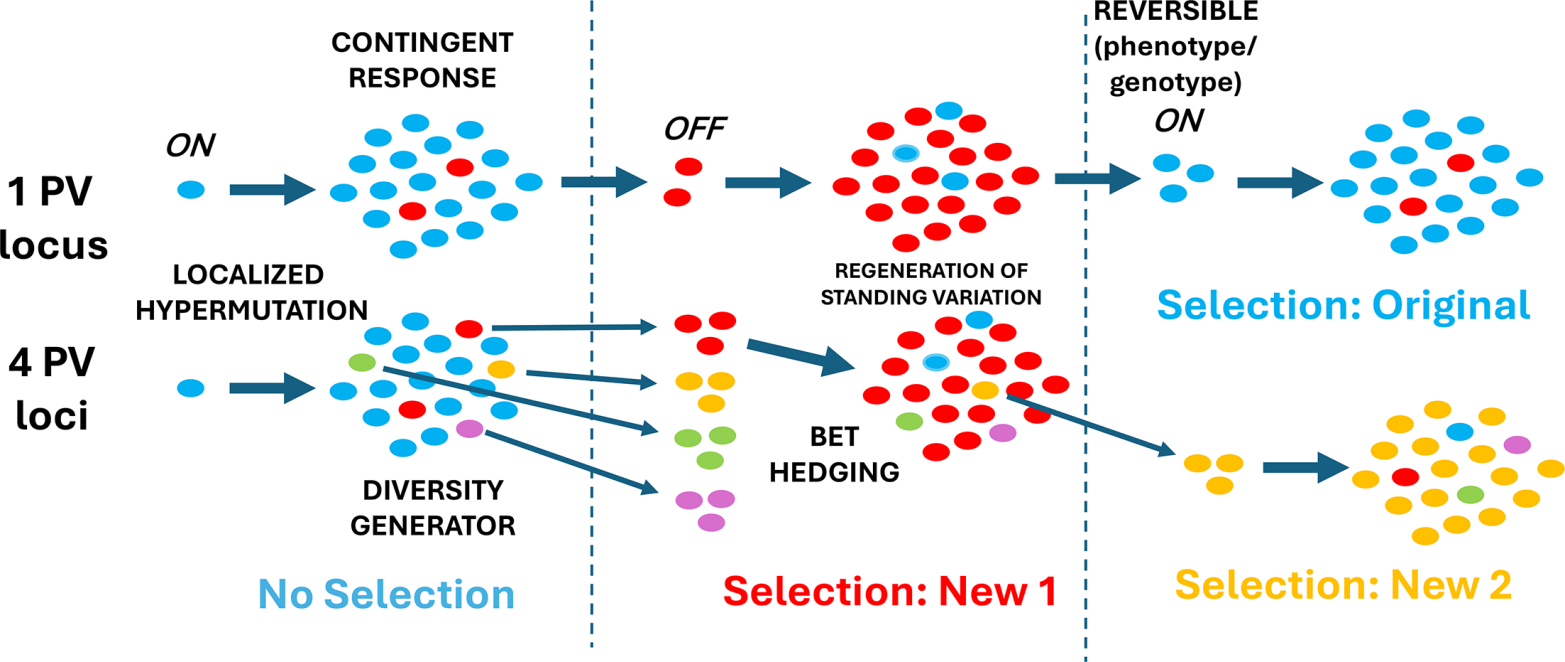
The major concepts of PV. This diagram shows bacterial strains with either one or four PV loci. A single cell is shown replicating to form a population in the absence of selection. High levels of switching in specific genomic regions generate phase variants in an alternate expression state (e.g. OFF if the starting phenotype was ON). This switching can occur through epigenetic or mutational processes, with the latter referred to as localized hypermutation (as illustrated). These PV loci are also diversity generators, a process that is amplified by having multiple PV loci in a single chromosome. The generation of variants prior to encountering the ‘new’ selection pressure results in pre-adaptation, the so-called contingent response, and bet-hedging, which emphasizes the preparation for encounters with adverse conditions. Note that when multiple PV loci are present, then there is bet-hedging for multiple selection pressures of which only two are depicted. Another key aspect is the reversibility of the genotype and phenotype; this is depicted as ON and OFF variants for the single-gene PV locus, with these revertants being able to survive reversion to the original selective conditions. A final key aspect of PV is the rapid regeneration of the original variants, or multiple variants if multiple PV loci, referred to as ‘standing variation’ in the wider genetic variation field, in the population after initial survival of the selective event.

In 1922, Andrewes [1] reported on the detection, with strain-specific antisera, of ‘mutually convertible types’ of *Salmonella* isolates in the absence of selection (later shown to be antigenically divergent flagella variants). A key feature was the observation of reversible switches between the two phenotypes during the passage of individual colonies. Subsequently, other authors reported numerous similar phenomena of reversible switches in surface antigens and transitions between colony morphologies. Edwards and West codified these observations stating that ‘phase variation’ (PV) had ‘a definite and limited meaning’ of reversible variation of surface antigens [2]. This terminology has retained its power and has only been broadened to refer to any gene that undergoes reversible switches of expression state at high frequency in the absence of selection. PV is, however, only a subset of a broader phenomenon of stochastic mechanisms for producing (epi)genetic variation in small populations [3]. We start, therefore, by setting out the broad principles of PV (Fig. 1) before moving on to the timeline of major discoveries and current trends in PV research.

Mechanisms for the generation of population heterogeneity have evolved in bacterial species due to the vulnerabilities of these single-cell organisms to selection arising from alterations in physico-chemical attributes of their external environments, competition and predation. Adaptation through alterations in gene regulation is a versatile fitness-maintaining tool but has limited applicability and flexibility due to constraints such as requiring a signal and a non-immediate response time and simultaneously affecting large proportions of the local population of cells. An alternate strategy is to generate a small number of variants prior to the encounter with the selective pressure (Fig. 1). Moxon *et al.* coined the term ‘contingency loci’ as an informative descriptor of this pre-selection, adaptive behaviour [3]. Other authors have likened this behaviour to the risk-spreading utilized during betting to reduce losses or increase the chances of winning [4]. In a biological sense, bet-hedging maximizes long-term fitness across several generations whilst incurring small fitness losses in any one generation. Both contingent and bet-hedging responses can be achieved by continually generating small numbers of variants that are maladapted to the current environment but will survive when a specific selective pressure is encountered. A significant extra benefit of PV is the rapid regeneration of the standing variation after exposure to the selective pressure so that the population is not vulnerable to a rapid reversion to the pre-selection conditions. These responses are critical aspects of the evolutionary success of the PV phenomenon and its underpinning mechanisms.

**Fig. 2.**
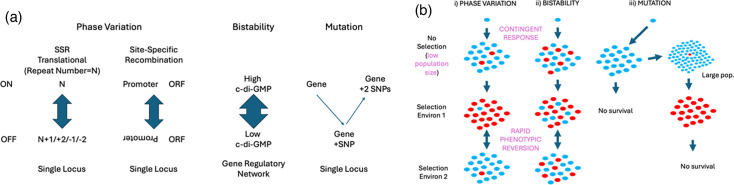
Stochastic generation of population heterogeneity via PV, bistability and ‘normal’ mutations (e.g. DNA replication errors). (a) Common mechanisms of each phenomenon. For PV, alterations occur in a specific locus and result in ON/OFF gene expression switches (two of the major mechanisms are shown here, namely, SSR and site-specific recombination, and the third, epigenetic, is shown in Fig. 3b). For bistability, alterations in the levels or activity of a transcriptional regulator, such as bis-(3’-’5’)-cyclic dimeric guanosine monophosphate (c-di-GMP), of a gene regulatory network result in switching between morphological variants or phenotypic states (e.g. motile/non-motile). For both PV and bistability, switches are reversible and occur at high rates. For mutation, variation occurs at low rates (e.g. by SNPs involving a change in a single nt or insertions/deletions of one or more nt), whilst ‘reversion’ is usually limited to compensatory mutations in another part of the same gene or another gene. (b) Differing population effects of these phenomena. A single bacterial cell generates a small bacterial population (e.g. 1×10^6^ c.f.u.) in the absence of selection (i.e. against the ‘blue’ cells) that contains genetic/phenotypic variants (the contingent response) for PV (i) and bistability (ii) but not for normal mutation rates (iii). Populations (i, ii) reversibly adapt to two specific selective environments due to the outgrowth of minor variants (i.e. ‘red’ or ‘blue’ cells) generated by reversible switching. In contrast, much larger populations (iii) are required for basal mutation rates to generate variants capable of surviving each change in the selective environment.

Mechanisms that facilitate stochastic generation of heterogeneous clonal populations can evolve in response to requirements for adaptation to frequently encountered, unpredictable, selective pressures. Critically, PV events are localised to specific genomic regions encoding gene products that are frequent targets of these selection pressures (Fig. 2). These pressures have acted on these genomic regions to increase the generation of genetic variation. As a result, PV loci have high switching rates with similar magnitudes for mutational, recombinatorial and epigenetic mechanisms. For example, mutation rates of the repeat tract in simple sequence repeat (SSR)-mediated PV loci are typically 100–10 000 times higher than basal mutation rates (as measured in mutations/bp/division), meaning that variants are reproducibly generated in small populations. This hypermutability provides a significant advantage over the intrinsic mutability of the majority of the genome as the latter requires relatively large populations for generating adaptive variants (Fig. 2). Moxon *et al.* coined the term ‘localized hypermutation’ to indicate the evolution of mutability in a specific genomic region [3]. This term distinguishes PV loci from the genome-wide increases in mutability arising from a mutator phenotype (e.g. due to disruption of mismatch repair genes). One significant benefit of localized hypermutation is the avoidance of the deleterious, off-target mutations generated by a mutator phenotype [5]. One apt descriptor of genomes with these adaptation-generating hypermutable or hyperswitching mechanisms is the ‘prepared genome’ [6]. This preparedness has been further amplified in some organisms by having multiple PV genes in a genome with these ‘diversity-generating’ systems, facilitating rapid adaptation to a wide range of selective pressures.

In a strict interpretation of the PV terminology, switching is between an ON and OFF expression state for a surface antigen in the absence of selection [2,7] (Table 1). Discoveries of other types of gene products with similar switching systems now mean that PV has a broader definition of referring to high-frequency, reversible switches in the expression of a specific gene or locus [8]. The phenomenon observed by Andrewes was for switching between two antigenic variants; this switch was subsequently shown to involve ON-OFF switching of one gene that is directly linked to the loss of repression of another gene, so fitting the strict definition [9]. Nevertheless, subsequent discoveries of shuffling between antigenic or genetic variants due to DNA sequence inversions of CDSs or promoters, termed shufflons, are included in the PV canon as no genetic information is lost in the phase variants [10,11]. This contrasts with the non-recoverable alterations in protein-encoding genetic sequences in loci subject to recombination-mediated antigenic variation (e.g. neisserial type IV pilus [12]) that are classified as contingency, but not PV, loci.

**Table 1.** Definitions of PV, contingency loci and bistability

Term	Definition
PV (strict interpretation)*	High-frequency, reversible switching (usually ON and OFF) of a surface molecule due to a mutational or epigenetic mechanism in a single locus
PV (broad interpretation)*	Any locus subject to high-frequency, reversible switching due to a mutational or epigenetic mechanism in a single locus
Shufflon	High-frequency, reversible switching between multiple, different gene alleles in a single locus
Contingency locus†	High-frequency antigenic or phenotypic variation due to localised hypermutation in a single locus but without a requirement for reversibility
Bistability	Bistable switches in a phenotype regulated by a transcriptional network

* See text for how these interpretations arose.

† PV loci are a subset of the contingency loci.

Bistability (see Fig. 2) is another widely observed population heterogeneity phenomenon that is associated with a range of phenotypes including some colony morphology transitions [13]. Bistability refers to switching between two complex phenotypic states where each state exhibits differences in the expression of multiple genes (Table 1). Transitions between bistable states are controlled by the interplay between transcriptional feedback loops and the intrinsic stochasticity of gene expression (see [13] for references). Bistability shares two key traits with PV: high-frequency transitions or oscillations between two different states and stochastic generation of low levels of adaptive variants in a clonal population. Despite these similarities, bistability is difficult to classify as either PV or a contingency locus due to the involvement of a transcriptional network for bistability as opposed to alterations in a single locus for the other phenomena. Table 2 highlights some of the initial discoveries in microbial research of both PV and bistable colony morphotypes as these phenomena were discovered in parallel and were difficult to separate in the absence of a mechanistic understanding. In later sections, we focus on PV due to the distinctive, locus-specific effects of these switching events.

**Table 2.** Timeline of major discoveries in PV

Year	Discovery	Reference
1922	First description of phase transitions in *Salmonella*	Andrewes [1]
1943	Definition of PV	Edwards and West [2]
1957*	PV in *Rickettsia*	Fiset [96]
1964*	L-phase variants in *Staphylococcus aureus*	Molander *et al.* [97]
1969	*Bordetella pertussis* PV and antigenic variation	Nakase *et al.* [98]
1975	Partial analysis of *Salmonella* flagella PV (Hin) locus	Enomoto and Stocker [18]
1977	Discovery of type I pili PV in *Escherichia coli*	Swaney *et al.* [99]
1979–1984	Definition of Hin locus *cis*- and *trans*-acting factors, activity of Hin recombinase	Silverman *et al.* [*9*]; Zieg *et al.* [100]; Silverman and Simon [101]; Kutsukake and Iion [102]; Bruist and Simon [103]
1981–1983	*E. coli* type I fimbrial PV, transcriptional control and locus	Eisenstein [104]; Freitag and Eisenstein [105]
1984	Opa PV in *Neisseria gonorrhoeae*	Black *et al*. [106]
1985–1987	Invertible promoter, FimB and FimE recombinases and IHF mediate *E. coli* type I fimbrial PV	Abraham *et al.* [107]; Klemmt *et al.* [108]; Dorman and Higgins *et al.* [109]; Eisenstein *et al*. [110]
1987	DNA modification controls *B. pertussis* PV	Goldman *et al*. [111]
1988	Recombination-mediated *Haemophilus influenzae* capsule PV	Kroll *et al*. [112]
1988	Invertible locus mediates pilin PV in *Moraxella bovis*	Marrs *et al*. [113]
1989–1994	Pap fimbrial PV requires DNA methylation by Dam and specific Lrp and PapI DNA-binding sites	Blyn *et al*. [114]; Blyn *et al*. [115]; van der Woude *et al*.[116]; Braaten *et al*.[117]
1989–1993	SSRs control PV in *Neisseria* and *Haemophilus influenzae*	Murphy *et al.* [118]; Weiser [119]; van Ham *et al*. [120]
1989	Frameshifts control *Bordetella pertussis* PV	Stibitz *et al*. [121]
1990–1991**	Combinatorial lipoprotein PV in mycoplasmas	Rosengarten and Wise [20]; Rosengarten and Wise [21]
1990	*Bacteroides fragilis* temperature-dependent phase shifts	Oyston and Handley [122]
1994	Definition of contingency loci and localised hypermutation	Moxon *et al*. [3]
1996	Multiple PV genes in *Haemophilus influenzae* genome	Hood *et al*. [23]
1998–2000	LOS antigen and genomic analysis of *Helicobacter pylori* and *Campylobacter jejuni* PV	Appelmelk *et al*. [123]; Saunders *et al*. [124]; Linton *et al.*[125] Parkhill *et al*. [24]
2000	Repeat number determines *Haemophilus influenzae* PV rates	De Bolle *et al*. [126]
2001	Comparative genomic analysis of neisserial PV genes	Snyder *et al*. [127]
2002	Mutators increase *Neisseria meningitidis* PV in epidemics	Richardson *et al*. [128]
2002–2003	Site-specific recombination and promoter inversion mediate *Mycoplasma* PV	Sitaraman *et al*. [22]; Flitman-Tene *et al*. [10]
2005	Phasevarion due to PV of restriction–modification	Srikhanta *et al*. [29]
2009–2010	Identification of invertase of *Bacteroides fragilis* PV system	Nakayama-Imaohji *et al*. [61]; Coyne *et al*. [129]; Patrick *et al*. [130]
2012	Repeat number determines *Campylobacter jejuni* PV rates	Bayliss *et al.* [131]
2012	*In vivo* escape of bacteriophages by PV in *Campylobacter jejuni*	Sørensen *et al.* [132]
2014	Six phase switches influence pneumococcal virulence	Manso *et al.* [47]
2018–2020	Phasomes: distribution of *Campylobacter* and *Neisseria* PV genes	Aidley *et al.* [54]; Wanford *et al.* [39]; Wanford *et al.* [65]
2019–2024	Invertons: widespread distributions	Jiang *et al.* [56]; Chanin *et al.* [57]
2024	Evidence for the evolutionary advantage of PV	Fernandez-Fernandez *et al*. [16]

*Switching in these systems may occur by bistability, not a classical PV mechanism.

* *Shuffling between genes as opposed to ON/OFF PV of single gene.

Damdeoxyadenosine methyltransferaseLOSlipooligosaccharide.Lrpleucine regulatory protein

The 100+ years of PV research has had three major strands – mechanisms, phenotypic responses and evolution. Mechanistic studies initially established general molecular mechanisms but have become ever more detailed, resulting in precise readouts of the determinants of PV switching rates. Studies of adaptive PV responses have focused on identifying the functions and selective pressures acting on both the ON and OFF expression states of phase-variable determinants, using model systems and epidemiological samples. Exploration of generic features through *in silico* models has simultaneously established a theoretical rationale for the evolution of different PV mechanisms [14,17]. As PV is now known to be widespread among bacterial species, answers to the how and why of PV are burgeoning.

## 100+ years of PV

The century-long PV timeline in Table 2 highlights the spasmodic discovery of new loci and mechanisms. In the two decades after the first observations by Andrewes in *Salmonella*, multiple observations of phase transitions of different types were reported in a wide range of bacteria – some detected as morphological changes in colony appearance or size and others with antigen-specific antibodies. Naturally enough, the mechanism of switching in the *Salmonella* flagellar locus was the first to be described in depth. Enomoto and Stocker [18] identified the invertible fragment responsible for switches in the expression. This was followed by a 10-year period identifying the site-specific recombinase, recombination site and the *cis*- and *trans*-acting factors that regulated the switching rate. Overlapping with these discoveries, other researchers were describing the similar, but more complex, dual recombinase system controlling PV of the *Escherichia coli* type I fimbria. Between 1985 and 1994, not only was a key mechanism of antigenic variation discovered in *Neisseria gonorrhoeae* (i.e. RecA-mediated alterations in the pilin filament protein [19]) but also the other major PV mechanisms: RecA-mediated recombination of *Haemophilus influenzae* capsule expression, hypermutable SSR and DNA methylation-based switching (as exemplified by the *E. coli* Pap fimbriae). The latter two mechanisms subverted prevalent dogmas that microbes would not contain microsatellites, due to the paucity of non-coding regions for these gene-inactivating sequences, or undergo epigenetic regulation.

The next significant milestone in PV biology was the discovery in 1990 of a multi-gene system for shuffling between allelic variants of a specific functional protein. This first example involved shuffling between lipoprotein variants in mycoplasmas that was subsequently shown to occur by site-specific recombination [10,20,22]. The advent of whole-genome sequencing (WGS) led to another major leap in PV research. *Haemophilus influenzae* was chosen as the first free-living bacterial species for the implementation of this technology in part to facilitate the search for PV genes [23]. This innovation identified 12 putative PV genes containing the characteristic SSR tracts known to mediate *Haemophilus influenzae* PV [23], representing the first evidence of multiple, functionally distinct phase-variable proteins in 1 organism. Rolling out of the WGS approach led to the identification of large SSR-mediated PV repertoires in a diverse range of species including *Neisseria meningitidis*, *Campylobacter jejuni*, *Helicobacter pylori* and *Treponema pallidum* [24,27]. WGS led to similar advances in the detection of recombination-mediated PV such as the identification of the multiple invertible genes controlling capsule switching in *Bacteroides fragilis* [28]. One of the surprises of these genomic studies was that type I and type III restriction–modification (RM) systems were subject to PV [23]. This led to the discovery in 2005 by Srikhanta *et al.* [29] of a gene expression regulon linked to switches in the expression of DNA methyltransferases that was termed the phasevarion. Thus, the PV of an RM system results in a genome-wide change in the methylation state of the DNA target sequences of this specific system and, through so far uncharacterized processes, alterations in the expression states of multiple genes (i.e. the phasevarion). Note that the phasevarion is an indirect consequence of RM PV. In contrast, epigenetic PV mediated by deoxyadenosine methyltransferase (Dam) is not due to phase-variable changes in the expression of this methylase but due to stochastic fluctuations in the interactions between certain regulatory proteins and Dam with their target sequences in the promoter of the phase-variable gene.

## Twin traits of PV: high switching rates and reversibility

The historical timeline highlights the discovery of PV in multiple, diverse organisms and of three distinct PV mechanisms, namely, SSR, site-specific recombination and methylation dependent (Table 2). Thus, PV has the hallmarks of parallel evolution as these disparate and unrelated mechanisms generate similar effects on gene expression, namely, high-frequency, reversible switches between two (or occasionally three) expression states occurring in a stochastic manner. As discussed above, Moxon *et al.* [3] elaborated the idea of contingency loci to explain multiple observations of high levels of locus-specific genetic or epigenetic variation. PV loci share the principal features of stochasticity and high switching rates with other contingency loci but can be distinguished by their reversibility, which is inherent in PV mechanisms. A complicating factor is that bistable phenotypes also exhibit high switching rates and reversibility. Three key aspects need to be addressed in order to define a locus or phenotype as PV rather than another type of bistability, contingency locus or low-level mutability: (1) switching is mediated by genetic or epigenetic variation in a single locus, (2) reversible switching and (3) a minimal switching rate (usually defined as a detectable number of phase variants in the absence of selection).

PV is clearly distinguishable from bistability by the association of phase-variable switches with alterations in a single locus, as outlined in the following sections. In order to emphasize these differences, we describe key aspects of bistability (for more details, see [13,30, 31]). Bistability refers to transitions between two states that occur at very high rates, observed as frequencies of 0.1–10% of variants per population. Bistable transitions are often between two morphotypes (e.g. L-forms and small-colony variants) and involve changes in the expression of multiple genes mediated by gene regulatory network (GRN) and often controlled by bis-(3’-5′)-cyclic dimeric guanosine monophosphate (c-di-GMP) signalling mechanisms [13,30, 32, 33]. First described by Rainey and Travisano [34] for switching between smooth and wrinkly spreader colony types in *Pseudomonas fluorescens*, these types of mechanisms are now known to be widespread [34]. A recent example is bistable switching in *Acinetobacter baumannii* between opaque and translucent colony morphologies. Switching occurs at a rate of ~0.25% and is controlled by a GRN and environmental signals [35,37]. The lack of association with a specific gene product, absence of localized hypermutability and involvement of reversible transcriptional regulatory mechanisms are major defining features that separate bistability from PV. Where some or all of these features are suspected or observed, we would suggest that bistability, rather than PV, terminology is the preferred descriptor.

The next consideration for defining PV is reversibility, which requires observation of both directions of switching in the absence of selection. Most of the initial observations of PV relied on colony immunoblotting with antigen-specific antibodies before being partially supplanted by reporter constructs and more recently by molecular methods. The older methods allowed for the detection of reversion as loss and gain of the expression of a gene product. However, at low switching frequencies, only one direction may be observed, and selection is often applied to detect reversion, which has the risk of amplifying rare variants by bacterial replication. A recent example is for the *mga* gene of group A streptococci [38]. Mutations in this gene, encoding a regulator of the surface M protein, occurred during laboratory culture and involved the shortening of a mononucleotide repeat tract from eight to seven cytosine repeats (i.e. 8C to 7C). However, reversion was only observed during infections of animals. The M protein is critical for immune evasion and adherence, meaning that infection imposes a very strong selection for reversion to the ON phenotype, with these reversion mutations occurring at an order of magnitude lower than for most PV genes. In the absence of evidence of spontaneous (i.e. non-selection driven) reversion, this locus cannot be categorized as having an evolved PV mechanism.

As the *mga* example illustrates, the rates of both directions of switching need to exceed a minimum threshold rate in order for a specific phenotype and/or locus to qualify as PV. Whilst there is no definitive lowest rate for PV, historic precedent has generally set the lower limit as that is detectable in the absence of selection. The classic methods for measuring PV rates in the absence of selection have a limit of detection of ~1 variant in 10^5^ colonies. For SSR PV, this lower limit has biological significance as around this limit difficulties arise in distinguishing evolved PV loci from ‘chance’ appearance of tracts due to the prevalence of short repetitive sequences in genomes [26]. For example, capsule switching of the MenB capsule of *N. meningitidis* has been associated with alterations in a 7C tract in the *siaD* gene. However, every meningococcal genome has ~40 loci with 7C/7G tracts, with many of these tracts expected to occur by chance due to the high prevalence of 6C/6G tracts in these guanine-cytosine-rich genomes [26,39]. Thus, there is no evidence for the evolution of a higher level of mutability in this *siaD* tract but rather an occasional (possibly beneficial) loss of MenB capsule expression due to the ‘accidental’ presence of this mutation-prone tract. Recently, Vargas *et al.* [40] identified multiple genes of *Mycobacterium tuberculosis* where indels in homopolymers were occurring at 100× the neutral substitution rate. However, these rates were an order of magnitude below those of most SSR-mediated PV genes, and none were shown to be reversible. This indicates that these loci cannot be termed as PV but could be described as mutation-prone adaptive variation.

PV switching implies the evolution of reversible phenotypic switching where both states provide fitness advantages during exposure to frequently encountered selective pressures. Broadening the PV definition to bistability or other non-hypermutable occurrences of adaptation, particularly where reversion of the underlying mutations or molecular events has not been demonstrated, has no identifiable benefit. Indeed, a broader definition will hamper attempts to tackle the difficult questions of how PV has evolved, what the relevant selective conditions are and identifying the benefits of PV to the bacterial population.

## New mechanistic insights and unknowns

Our historical timeline celebrates significant advances in understanding the mechanistic basis of PV switching and regulation of phenotypes (Table 2). As detailed reviews of the three major switching mechanisms, namely, SSR, site-specific recombination and epigenetic, are available elsewhere [8,41, 42], we have focused on recent findings. Advances in understanding site-specific recombination-based PV have continued due to the combined implementation of structural and biochemical approaches and innovations in sequencing technologies (see below). A novel regulatory insight is the intersection between PV and c-di-GMP signalling. The combination of stochastic elements of PV and the bistable regulatory pathway of c-di-GMP signalling is predicted to enhance adaptation to a range of environments. Mechanistic advances for other modes of PV have been more limited, and major gaps remain in our understanding of the determinants of switching rates in these systems.

*Salmonella* flagella PV still fascinates researchers. In 2004, Dhar *et al.* [43] used a combination of structural studies, genetics and *in vitro* recombination assays to develop a working model for how the serine recombinase (invertase) generates a dsDNA break at the *hix* switch site. In the supported model, a tetramer of Hin bound to *hix* was the active recombination complex with a ‘tetrameric swivel’ rotational mechanism predicted to allow for DNA strand exchange. These results were extended to characterizing contributions of specific aa in Hin [44] and the bacterial histone-like protein Fis [45] to recombination rates. Additional secondary regulation by other molecules is now beginning to be uncovered; for example, a conserved CsgD regulator appears to degrade *hin* DNA segments [46].

New sequencing technologies have facilitated the characterization of rates and mechanisms of recombination-mediated switching. Initially, the technical challenge of analysing multi-invertible *hsdS* PV loci was met for the pneumococcal SpnIII R/M system [47] by quantifying DNA sequence variant frequencies with a fluorescent PCR-based GeneScan assay. Rates of switching between multiple HsdS sequence variants were quantified and shown to depend on a site-specific recombinase, encoded within this locus, and specific inverted repeat sequences. As these methods are laborious, the report by Roodsant *et al.* [48] of quantification of inversion-mediated switching of a type I RM system of *S. suis* by PacBio and Oxford nanopore sequencing is likely to enhance the evaluation of switching rates and determinants for these complex recombinatorial systems. Another application is for PV events involving insertion sequence (IS) elements. These elements are typically associated with low-frequency unidirectional, non-PV switching events. However, building upon prior data obtained with long-read next-generation sequencing (NGS) [49], Lowrey *et al.* [50] showed that an IS-bounded element of 209 kbp undergoes copy number variation in *Burkholderia thailandensis*. Mutations were reversible and controlled by RecA acting on homologous ISs flanking the variable region. Variations were detectable in colonies so are probably occurring at a high stochastic rate consistent with PV. These NGS approaches are widely applicable to PV inversion mechanisms and may facilitate analyses of switching rates in the absence of selection and evaluation of control mechanisms.

**Fig. 3.**
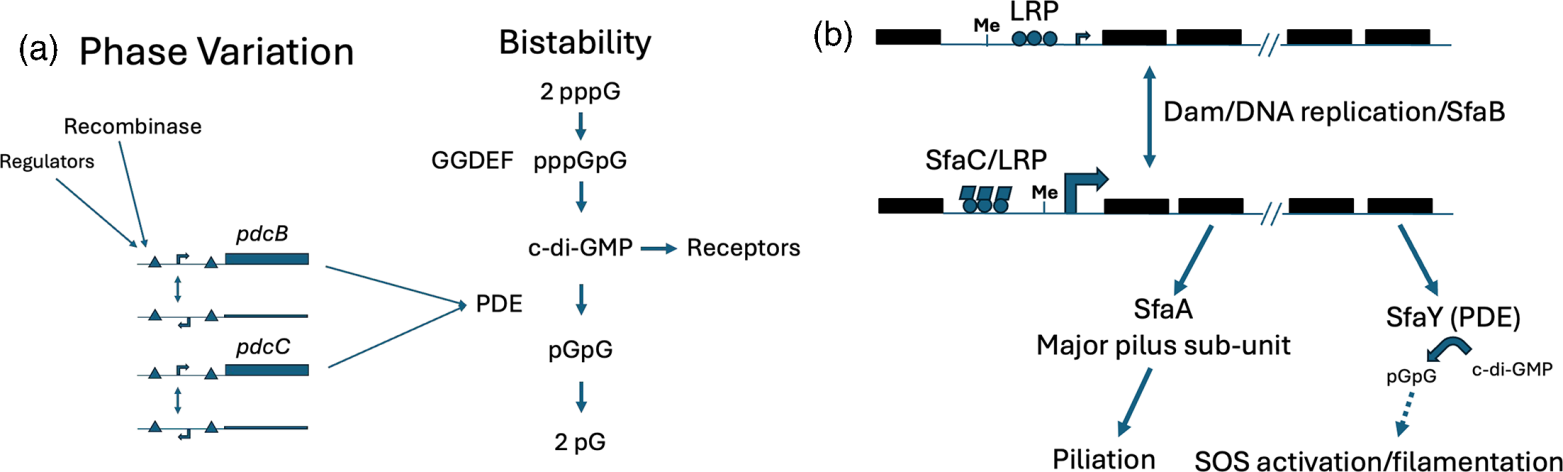
Links between PV and c-di-GMP signalling. (a) Reyes Ruiz *et al.* observed that PV by site-specific recombination of the promoters of two PDE-encoding genes in *Clostridioides difficile* results in heightened turnover of c-di-GMP molecules [51]. Switching of either or both the PDE genes into an ON state altered the regulation of pathways and phenotypes controlled by c-di-GMP. (b) Zlatkov *et al.* [53] found a PDE-encoding gene within the *sfa* fimbrial operon in *E. coli*. Epigenetic PV of this locus results in concomitant switching ON of PDE expression, resulting in c-di-GMP turnover, SOS response activation and filamentation. These examples show how PV can add further layers of stochasticity and plasticity to already complex regulatory systems.

Another area of advance has been identifying and dissecting the intersection of regulation by PV and bistability mechanisms (Fig. 3). This is exemplified by *Clostridioides difficile* where levels of the signalling molecule c-di-GMP are partially dependent on the expression of two phosphodiesterases (PDEs), encoded by genes *pdcB* and *pdcC.* Both of these genes have invertible promoters and hence are subject to PV [51,52]. The expression of these genes correlated with changes in c-di-GMP levels and resulted in concurrent impacts on sporulation, motility and biofilm formation. Zlatkov *et al.* [53] provided another example of a link between PV and c-di-GMP bistable regulation. In a small set of atypical, meningitis-associated isolates of *E. coli*, a range of variable phenotypic traits, including metabolic profiles, were found to be partially dependent on a PDE that was subject to epigenetic PV as part of the epigenetically regulated *sfa* fimbrial operon (Fig. 3). These examples raise questions about the advantages of adding phase-variable switching to the complex phenotypic variation, resulting from a bistable GRN. One possibility is that further increasing population heterogeneity generates additional population sub-groups and allows for more sensitive responses to environmental selective pressures or widens the gradient of responsiveness.

After 100+ years of research, observations of PV are still dominated by the three major mechanisms (as exemplified in Figs2a  3b). This viewpoint may change through the application of novel sequencing technologies to a widening spectrum of organisms and phenotypes. These approaches may demonstrate that, for example, IS-dependent switching is commonplace or identify novel mechanisms for achieving PV switching.

## The phase-variable repertoire

Despite the 100+ year history, phase-variable loci are continually being discovered in both old and new organisms. Major boosts in finding loci have come from the advent of new technologies such as long-read sequencing methods. Whilst not intended to be comprehensive (and, as outlined above, subject to arbitrary distinctions), Table 3 provides a view of the number of species where PV has either been described in the literature or discovered by searches for specific mechanisms. The limited overlaps between these datasets indicate our inadequate knowledge of the PV phenomenon. Additionally, many of these organisms may only contain one or two PV loci, whereas species with multiple PV loci are still relatively uncommon. We highlight a few examples of approaches to finding the distribution of known mechanisms of PV loci.

**Table 3.** Numbers of bacterial genera and species containing putatively phase-variable loci

Dataset	Genus (species)	Overlap with abstracts	Overlap of PF/LSSR
Abstracts*	47 (76)	–	–
PhaseFinder†	133 (231)	25 (21)	–
Long SSRs‡	153 (246)	32 (34)	35 (33)

* Abstracts were obtained from PubMed for publications with titles containing ‘PV’ or similar phrasing (e.g. phase variable) with species names extracted from the text using an R script.

† PhaseFinder (PF) is an algorithm, as published by Jiang *et al.* [56], that was used to search 54 875 bacterial genomes for invertible intergenic regions flanked by repeats [56].

‡ Long simple sequence repeats (LSSR), as published by Mrazek *et al.* [133], are defined as SSRs of repeat length *k* whose total length exceeds a cutoff derived from a random model, which reproduces key genomic sequence metrics. This latter dataset was generated from a search of 378 prokaryotic genomes for these repeats [133].

Over the last two decades, NGS approaches have revolutionized the identification of recombination and SSR PV mechanisms (as exemplified in Table 3). Identification of recombinatorial switching requires careful analysis of raw sequence data to extract inverted sequences, whilst for SSRs, the critical step is the definition of hypermutable repeat numbers not expected to occur based on genome composition alone. Both methods may benefit from high frequencies of switching occurring during the preparation of genomic DNA for sequencing [24,47]. Advances in bioinformatic tools have underpinned the detection of PV loci/mechanisms in a diverse range of bacterial species with multiple competing approaches being described [54,55]. Comparison of methodologies and definitions may be a critical part of this process as the PhaseFinder (4686 invertons/54 875 genomes) and PhaVa (4662 invertons/30 000 genomes) methods for finding invertible elements have found differing numbers of loci [55,57].

Unlike SSR- and site-specific recombination-mediated PV, the discovery of epigenetic PV mechanisms in novel loci is lagging behind. This is despite technical advances for the detection of nt methylation patterns by PacBio or nanopore genome sequencing approaches. The key problem is that not all methylation patterns will result in PV [58]. Epigenetic regulation can be found if details of the protein partners of the methyltransferase involved in the epigenetic mechanism are known. Thus, multiple glycosyltransferase operons for O-antigen modification across *Salmonella* species were predicted to undergo epigenetic PV based on the presence of Dam sequences/OxyR DNA-binding motifs known to control PV [59]. Finding novel epigenetic loci should be feasible by correlating variable methylation with RNA-Seq gene expression data but is fraught with difficulties. The alternative approach of the identification of relevant phenotypic variants and the confirmation of causal links between DNA methylation states and gene expression changes is significantly more time-consuming.

Historically, PV events and loci have been related to surface structures, directly or indirectly, and particularly pili, fimbriae, capsules or lipopolysaccharide. This viewpoint has been challenged by data from NGS with rapid expansions to the scope and species. Most notable is the recent description of PV of PDEs that affect c-di-GMP levels (see the section ‘New mechanistic insights and unknowns’) and the signal transducer, CmrRST, in *Clostridioides difficile* [60]. Another notable finding is the roles of PV in nutrient acquisition with the association in *Bacteroides* species of invertase-mediated phase-variable genes with the utilization of host-derived glycans [61]. There have also been expansions to novel organisms such as the identification of SSR-mediated PV of a sialidase in *Gardnerella* spp. [62]. Long-read NGS technologies have also allowed the discovery of inversions of larger DNA segments, up to several 100 bp, that can generate PV-like phenotypic effects. Two examples are the inversion of a 600 kbp fragment in *Lactococcus lactis* subsp. lactis bv. diacetylactis S50 that regulates a spectinomycin-resistant slow-growth phenotype [63] and large-scale inversions in antibiotic-resistant small-colony variants of *Staphylococcus aureus* [64]. Whilst it is unclear if inversion rates for longer fragments occur at frequencies commensurate with PV, novel findings are likely to continue as NGS datasets accumulate.

Another output of ever-increasing genomic datasets is the exploration of complete sets of phase-variable genes within genomes (referred to as the ‘phasome’) with several host-restricted species containing ten or more PV genes. As PV genes are often non-essential, phasomes are expected to vary between strains of a species and/or genus. The development of specific SSR-mediated PV detection tools has led to genomic predictions of phasomes for multiple lineages of *N. meningitidis* and several species of *Campylobacter* [39,54, 65]. Phasome size depends on repeat number cutoffs, which vary by repeat unit length. One finding is that a lineage or genus has a core phasome of conserved PV genes likely representing evolutionarily stable functional genes. Contrastingly, high strain-to-strain variability is also observed, reflecting a lack of strong stabilizing selection acting on many PV genes. No specific patterns to core phasome functions have emerged other than the conservation of repertoire subsets encoding phase-variable outer membrane proteins or modifiers of surface structures.

Whilst this brief overview highlights the power of genomics and NGS technologies, phenotypic methodologies are still delivering critical advances. Taking a novel approach, Sørensen *et al.* [66] demonstrated that PV occurs in phages [66]. These authors showed how the effects of SSR-mediated switches in the expression of receptor-binding protein (Rbf) could be blocked with Rbf-specific antibodies, leading to the loss of a dual-plaque morphology and the production of a single plaque type.

The ongoing description of novel systems and phase-variable repertoires opens a window into several unanswered questions. One major question concerns the apparent restriction of multi-locus PV genomes to pathogens. This association may be due to high, but intermittent, levels of selection during colonization of hosts but more prosaically may result from biases in genomic databases towards bacterial pathogens and commensals. Evolutionary explanations for the PV phenomenon may be informed by the future expansion of PV locus distributions across microbial, archaeal and phage phylogenies.

## Phenotypic impact of PV: single and multi-gene effects

Surface proteins/structures or RM systems as predominant functions for PV loci have underpinned the idea that host–microbe or phage–microbe interactions are major drivers of PV-mediated adaptation. As a result, most experimental testing has focused on the exploration of these selective pressures. One approach involves the use of epidemiological samples from natural or model infections to detect and quantify PV switches. Another approach is to test the effects of specific selective pressures on one or more of the PV states of specific loci in either *in vitro* or model systems. One of the under-researched aspects is the benefits of rapid access to the extraordinary diversity produced by multi-gene systems. Thus, organisms with 12 or 20 PV genes capable of ON-OFF switching can generate 4096 or 1 048 576 different combinatorial phase variants, respectively. These combinatorial variants, termed phasotypes, are a powerful aspect of PV diversity generation but are a technically challenging aspect to study. Thus, the literature on the adaptive benefits of PV is extraordinarily broad, reflecting the experimentation on a rich diversity of PV mechanisms, PV functional groups and organisms. Before presenting a few recent exemplar publications, we discuss the critical elements of PV experimentation.

Exploration of the phenotypic impact of PV should consider the following basic tenets: one, there should be selection for both the ON and OFF states (or multiple states for more complex systems); two, observed switching rates should be more advantageous than low or no switching; three, a population is required that is of sufficient size for generation of variants (and is likely proportional to the switching rate); four, biological replicates of selection experiments should result in different outcomes due to stochastic generation of variants in starting populations. For multi-gene PV systems, experiments should also account for the potential impacts of combinatorial switching. For experimental systems, there is usually a focus on the selection of a single PV state in a small number of experimental repetitions and occasional comparison to mutants where the locus is fixed into one specific state. For observational studies, data are collected on PV switches over time, spatially or both. These studies are often limited by the lack of knowledge about the selective forces acting on the PV states, sampling issues and limited independent repetitions. Retrofitting and exploration of data relative to the basic tenets through *in silico* modelling can reduce the limitations of experimentation, and hence, modelling has become critical for deriving the maximum benefit from PV studies.

An important experimental approach in PV phenotypic research is the generation of mutants locked into one expression state. This approach reduces complexity and becomes progressively more important, but difficult, as systems increase in complexity from single to multi-genic PV systems. Further challenges arise in systems where the expression of multiple PV systems is coordinated such as for PV of *E. coli* fimbrial operons (reviewed in [67]). Using locked ON mutants, Snyder *et al.* [68] showed that PV of type I fimbriae was important during infections of animals by uropathogenic isolates. This experiment did not, however, demonstrate that PV per se was required for virulence, as mutants with differing PV rates were not evaluated [68]. This illustrates one of the key challenges in showing that PV itself is a virulence factor. In contrast, locked mutants for the canonical *Salmonella* flagella PV system have facilitated a paradigm change from a view of a simple antigenic switch to an infectivity switching system. Thus, constitutive expression of FliC results in higher infectivity as compared with constant FljB expression due to changes in the adhesion and motility phenotypes [69, 70]. These advances have been underpinned by carefully designed mutants, structural studies and testing of a multitude of experimental conditions. Similar studies have been performed on the more complex shufflon of *Mycoplasma agalactiae* variable lipoproteins (Vpmas). Sommer *et al.* generated mutants locked into the expression of specific Vpmas, by deletion of the XerC recombinase, and showed variable efficacy of killing by bactericidal antibodies raised against specific lipoprotein variants [71]. Finally, in a major achievement, deletion of all 11 phase-variable *opa* genes was achieved for 1 *N. gonorrhoeae* strain [72]. By constitutively expressing one Opa protein in this strain, Alcott *et al.* demonstrated that neutrophils more efficiently internalized gonococci expressing certain Opa protein variants, underscoring how Opa PV is critical for gonococcal evasion of neutrophil clearance [73].

Monitoring PV switches in model systems, animal infections or clinical samples has provided insights into the biological relevance of PV. This work has seen a move from focusing on single PV gene effects to determining the extent of variation and phenotypic benefits produced by multi-gene systems. One recent example focused on one of several *Haemophilus influenzae* PV genes encoding lipooligosaccharide biosynthesis genes. Using chinchillas as a tractable model of otitis media infections and nasopharyngeal colonization by non-typeable *Haemophilus influenzae*, Wills *et al.* demonstrated that acetylation of the lipooligosaccharide in *oafA* ON variants inhibited lipooligosaccharide-specific antibody production during infections and protected against opsonophagocytic killing in *in vitro* assays [74]. Studies of multi-gene systems have included two on the zoonotic pathogen, *Campylobacter jejuni*. Kim *et al.* tracked switching in 19 of the 23 SSR-mediated phase-variable genes of *Campylobacter jejuni* strain NCTC11168 and found associations between switches in 2 loci and colonization and disease in mice [75]. Wanford *et al.* analysed six-gene phasotypes (e.g. ON-OFF-ON-OFF-OFF-ON) of another *Campylobacter jejuni* strain to show that very small non-selective bottlenecks occurred during experimental infections of chickens [76]. This latter study is an example of the power of PV analysis for detecting shifts in population structures during infections. Similar studies are ongoing with a variety of *Bacteroides* strains where multiple PV genes in each genome control capsule structures, polysaccharide metabolism and RM systems. Recent studies have found associations between inversion and expression states of subsets of these PV genes with inflammatory bowel disease that are in part driven by the inflammatory conditions and presence of bacteriophages [77,78]. Pathogenic *Neisseria* are another important group of organisms with multiple PV loci. In a study of natural, persistent meningococcal carriage, Green *et al.* [79] measured SSR-mediated PV of ~15 genes in 25 carriers during longitudinal colonization and obtained evidence for compensatory PV of Opa proteins in some carriers [79]. Epidemiological investigations of PV in human volunteers or clinical isolates are challenging to set up and hence are still quite rare; these studies are, however, rapidly advancing our understanding of how PV contributes to clinically relevant bacterial infections.

Starting with the discovery of the phase-variable *Haemophilus influenzae* RM systems in 1996 [23], one of the major paradigm shifts in PV biology has been the linkage of phase-variable switches in RM expression to the regulation of gene expression. Bioinformatic analyses of genomic data have detected phase-variable RM systems in a large and diverse range of bacterial species and a variety of switching mechanisms including SSR-mediated PV of a single methyltransferase and multi-allelic, recombinatorial PV of DNA target recognition domains of type I RM systems [80]. Switching of these RM systems generates variation in genomic DNA methylation patterns between PV variants. Recognizing the importance of these observations, Srikhanta *et al.* [29] coined the term phasevarion to describe their observations of associations between specific PV-controlled DNA methylation states and global changes in both transcriptomes and phenotypes [81]. New NGS technologies have facilitated further discovery of phasevarions by allowing for rapid identification of the DNA methylation target sites and their regulons. Intriguingly, stable linkages have been observed in *N. meningitidis* between specific target recognition domains of phase-variable methyltransferases, and hence DNA modification patterns, and specific hypervirulent lineages, which is indicative of selection for fitness enhancements, resulting from the expression of a particular phasevarion [82]. Problematically, the mechanism linking PV of RM methyltransferase activity to changes in the expression of specific genes and phenotypes has not been clearly demonstrated. In the absence of this data, it is not clear if phasevarions are an evolutionarily stable strategy or whether other fitness benefits, such as phage resistance, may have driven the evolution of these phase-variable RM systems [83].

This snapshot provides a window into the successes and frustrations of PV experimentation and obtaining evidence of the adaptive benefits of PV in real-world systems. Indeed, an outstanding challenge in PV research is to prove that the PV mechanism (i.e. the ability to switch) is a virulence factor. In 2024, Fernández-Fernández *et al.* [16] made significant headway using a model whereby *Salmonella enterica* strains were exposed to forward (by phages) and counter (by serum) selection. The authors found that PV of the phage receptor, controlled by PV of the *opvAB* operon, was advantageous over fixed resistant strains in fluctuating but not constant conditions [16]. Mathematical modelling indicated that the generation of heterogenous populations by PV was beneficial specifically in fluctuating environments. Evidence for the benefits of switching and for forward and counter-selective pressures acting on specific PV loci should be amenable to well-designed testing regimes. Widespread implementation of ‘best practice’ from the canonical systems of the first 100+ years of PV research is required to reveal the selective benefits and intricacies of this premier adaptive strategy.

## Real-world data and the ‘not looking’ gaps: where lies the big impact of PV?

Despite major advances in understanding how PV contributes to bacterial survival and virulence strategies, this knowledge has not been routinely applied to improving plant, animal and human health through new therapies, preventive measures, diagnostics or other impacts. However, potential ‘real-world’ impacts of PV research are accumulating in the infectious diseases field. A key starting point is the frequently cited rationale for the evolution of PV: ‘evasion of the immune system’. This viewpoint is based on extensive evidence of phase-variable switching of antigenic surface structures (e.g. proteins or carbohydrates), leading to the loss of recognition by antibodies. Expanding on this concept, we highlight some thought-provoking studies on the impacts of PV on infection, vaccine design and antimicrobial resistance (AMR).

The WHO priority list of bacterial pathogens includes several organisms with extensive PV repertoires [84]. Vaccines are effective mechanisms for preventing bacterial disease and a viable option for limiting AMR and combatting disease by antibiotic-resistant isolates [85]. Irrespective of vaccine type, awareness of potential impacts from phase-variable structures should be integrated into the design and preparation of vaccines as this may enhance efficacy. Green *et al.* [86] addressed this knowledge gap by examining coverage by one component of the recently licensed 4C-MenB vaccine, a successful multi-component, recombinant vaccine targeting *N. meningitidis* serotype B strains [86,87]. One of the recombinant proteins in 4C-MenB, NadA, is subject to PV [88]. By correlating a determinant of protection (i.e. positive bactericidal threshold in the Meningococcal Antigen Typing Scheme) to NadA expression levels, as inferred by the PV signature (nt repeat tract length), Green *et al.* provided strong evidence that strain coverage of the 4C-MenB vaccine may be impacted by lower NadA expression due to PV of this gene [86].

Rational design is the latest approach in vaccine development. A *Streptococcus pneumoniae* vaccine with comprehensive serotype coverage is of considerable health interest. The discovery of phasevarions in pneumococci led Phillips *et al.* to consider how PV impacts vaccine design [89]. The expression of several putative proteinaceous vaccine candidates was perturbed by the methylation specificity of SpnIII, a ‘master’ phasevarion regulator [89]. The authors concluded that proteins from these phasevarions should not be included in a rationally designed vaccine. An alternate view, based on the principles of the successful Men4B vaccine, is that phase-variable proteins can make valuable contributions to a multicomponent vaccine, since PV often impacts gene products that are critical to infection processes [87,88]. Taking this a step further, knowledge of PV-mediated immune evasion has been exploited in the ‘evolutionary trap’ vaccine design as proposed by Diard *et al.* [90]. The underpinning idea is that pathogen escape from vaccine-induced clearance should only occur if it leads to reduced virulence. This concept arose from the authors’ study of a vaccine comprising all the immunodominant lipopolysaccharide O-antigens for *Salmonella enterica* subsp. enterica serovar Typhimurium. Several of these antigens are glucosylation variants that can arise due to PV of *gtr* operons [90]. Upon experimental infection, escape of vaccine-induced immune clearance occurred specifically through shortening of the O-antigen. These escape variants, however, were highly attenuated [90]. Critically, setting the evolutionary trap was feasible because the number of PV O-antigen variants in this serotype was limited and known [59]. This approach may be feasible for other species and phase-variable antigens (i.e. surface proteins or other lipopolysaccharide epitopes) where there is extensive knowledge of how PV impacts both immune evasion and virulence.

Diagnostics is another area where knowledge of PV may be exploited. Several classical serotyping methods rely on the reactivity of antibodies against surface antigens, some of which are affected by PV. Standard *Salmonella* serotyping relies on antibody recognition of O-antigen modifications with some isolates being termed ‘untypeable’ due to the target antigen being in the OFF PV state (unpublished, Van der Woude and Davies). In a 2023 study, analysis of the outer membrane protein OpiA (HopA) in *Helicobacter pylori* isolates from patients detected correlations between *opiA* PV state and occurrence of gastric cancer and patient outcome/risk factors [91]. This finding suggests that *oipA* PV state analysis could be developed as a prognostic tool for personalized predictions of *Helicobacter pylori* infection outcomes and used to identify optimal therapies. In another important pathogens, *Mycobacterium tuberculosis*, the PV state of GlpK, a kinase important in glycerol metabolism, was associated with transient drug tolerance [92] and predisposed populations towards the development of antibiotic resistance [93]. Finally, correlative links between host immunological responses and PV states of *Bacteroides fragilis* polysaccharide A derived from metagenomic samples may provide clues to the causation of inflammatory bowel disease [78]. These exciting examples of PV impacting disease outcomes and diagnostics lead us to posit that accounting for PV in the screening of infectious diseases is imperative and ultimately may support the development of improved, personalized therapies.

The potential impacts of PV on antibiotic use should be incorporated into the development and application of new and old treatments. Concerningly, evidence is accumulating that some AMR-conveying genes are subject to PV. Utilizing PhaseFinder (see above), Jiang *et al.* ] found evidence for direct effects of PV on AMR [56]. Specifically, in *Bacteriodales*, a group of beneficial human gut commensals, phase-variable glycan synthesis loci were found to contain putative AMR genes. Concerningly, these loci were also present on integrative conjugative elements that could facilitate the horizontal spread of these PV-linked AMR genes [94]. PV may impact phage therapy, a strategy for combatting AMR that is undergoing a significant resurgence in interest. Phage adsorption to the bacterial cell can be impacted by PV-mediated loss of expression of the phage (co)-receptor, or modification of this structure with a phase-variable moiety [16,66, 95]. These types of PV events could reduce the effectiveness of a therapeutic phage. Conversely, a benefit may arise if selection for PV-mediated phage evasion results in reduced virulence of the pathogen. Thus, the complex interplay of phage, host and immune response to phase-variable surface factors may impact the long-term success of phage therapy regimes. Taken together, these examples illustrate how new, targeted PV experimental data are needed to inform improved diagnostics and therapies for infectious diseases.

This section has illustrated how negative consequences may arise from ‘ignoring’ PV or, conversely, how PV data may inform screens for novel vaccine or drug targets. A summary of ideal PV assessment strategies is beyond the scope of this review but might involve using genomic or epigenomic data to detect signatures of PV facilitating predictions of PV-mediated immune evasion or identification of a pathogen’s ‘Achilles heel’ for survival. The overall focus on pathogens affecting human and animal health reflects current knowledge, but we anticipate filling knowledge gaps for bacteria living in other stressful environments (e.g. ocean and soil) where PV may have evolved as a coping strategy and hence could inform, for example, global warming or food production interventions.

## Concluding remarks

Over a hundred years ago, PV was first observed and described. Since then, there has been a steady increase in publications on this subject, and it is heartening to see a resurgence in awareness of phase-variable mechanisms and phenotypes. This resurging interest in PV has been driven in part by new approaches, with the current range of genomic and bioinformatic tools improving the detection of the PV hallmarks of minor, stochastic changes in genome sequences. Exciting discoveries, of which only a handful could be presented here, in a broad range of species have yielded new insights into how PV has affected the evolutionary success and survival of bacteria. The increasingly integrated approaches combining mathematical modelling, evolutionary theories and host biology are continuing to expand our appreciation of population biology within microbiology. During this shift from a focus on single model organism biology to population biology (cooperation and competition) and host–bacterium interaction, it is imperative that PV concepts are not forgotten. Whilst hard to study or account for experimentally, PV will continue to yield valuable discoveries and translationally important outcomes for modern basic and applied microbial research.

## Abbreviations

AMR: antimicrobial resistance
c-di-GMP: cyclic dimeric guanosine monophosphate
Dam: deoxyadenosine methyltransferase
GRN: gene regulatory network
IS: insertion sequence
NGS: next-generation sequencing
PDE: phosphodiesterase
PV: phase variation
Rbf: receptor-binding protein
RM: restriction–modification
SSR: simple sequence repeat
WGS: whole-genome sequencing
